# Spatial modelling of the shared impact of sexual health knowledge and modern contraceptive use among women with disabilities in Africa

**DOI:** 10.1186/s40834-025-00349-4

**Published:** 2025-02-28

**Authors:** Obasanjo Afolabi Bolarinwa, Clifford Odimegwu, Aliu Mohammed, Ezra Gayawan

**Affiliations:** 1 Demography and Population Studies Programme, Schools of Public Health and Social Sciences, University of the Witwatersrand, Johannesburg, South Africa; 2 Department of Public Health, York St John University, London, UK; 3 Department of Health, Physical Education and Recreation, University of Cape Coast, Cape Coast, Ghana; 4 Department of Statistics, Federal University of Technology, Akure, Akure, Ondo State Nigeria

**Keywords:** Spatial modelling, Shared impact, Sexual health knowledge, Modern contraceptive use, Women with disabilities, Africa

## Abstract

**Background:**

Women with disabilities remain highly vulnerable to sexual and reproductive health problems, particularly in sub-Saharan Africa (SSA), where their sexual and reproductive rights, such as access to sexual health information and contraception, are often neglected. This study investigated the spatial patterns of the shared impact of sexual health knowledge and modern contraceptive use among women with disabilities in Africa.

**Methods:**

We used the most recent Demographic and Health Survey (DHS) data involving 16,157 women with disabilities from ten African countries for this study. The data were analysed using both spatial and Bayesian inference to account for the shared component model patterns between sexual health knowledge and modern contraceptive use among women with disabilities while accounting for factors unique to each outcome. Bayesian inference via the Integrated Nested Laplace Approximation (INLA) was used for implementation. Priors for shared effects ​were set as log-normal distributions, while Gaussian priors were assigned to fixed effects. Intrinsic Conditional Autoregressive (ICAR) priors modelled spatial dependencies between districts, introducing spatial autocorrelation based on shared boundaries. Penalised Complexity (PC) priors controlled precision parameters to balance model complexity.

**Results:**

The study revealed low sexual health knowledge (ranging from 3% in Nigeria to 27% in Uganda) and modern contraceptive use (ranging from 1% in DR Congo and Chad to 27% in Uganda) among women with disabilities across the countries surveyed. The spatial patterns showed diverse intra-country and inter-country disparities of sexual health knowledge and modern contraceptive use among the women, with lower shared impact observed in Mauritania, Nigeria, Uganda, Chad, and DR Congo relative to Kenya, Malawi, Mali, South Africa, and Rwanda. Factors that influence sexual health knowledge and modern contraceptive use among women with disabilities include education, marital status, place of residence, community literacy level, community socio-economic status, and age.

**Conclusions and recommendations:**

Sexual health knowledge and modern contraceptive use among women with disabilities in Africa remain low, albeit with varied intra-country and inter-country spatial disparities. Therefore, spatial areas with low sexual health knowledge and modern contraceptive use should be given more attention when implementing measures to promote the use of modern contraceptives among women with disabilities. Promoting sexual health knowledge and modern contraceptive use among women with disabilities in Africa could significantly contribute towards the realisation of the 2030 Sustainable Development Goal agenda of “leaving no one behind”.

## Background

Globally, women with disabilities are highly exposed to sexual and reproductive health problems due to systemic barriers and discrimination with respect to their sexual and reproductive rights [1, 2]. Despite the various legal frameworks, protocols, and agreements at national and international levels, women with disabilities continue to experience various forms of sexual and reproductive health violations, including forced sterilisations, forced abortions, lack of access to contraceptive choices, forced marriages, limited access to sexual health information, sexual violence, and sexual abuse [1, 3, 4].

These issues contribute to the increased risk of sexual and reproductive health problems among women with disabilities. For instance, lack of access to modern contraceptive choices and limited sexual health information has been associated with an increased risk of unintended pregnancies, unsafe abortions, and sexually transmitted infections (STIs), including HIV, among women with disabilities [5–7]. Yet, there is a lack of attention to the sexual and reproductive health problems of women with disabilities, particularly in sub-Saharan Africa (SSA), although women with disabilities have the same sexual and reproductive health needs as those without disabilities [8].

Sexual health knowledge is important in equipping individuals with the right information, skills, and attitudes to make informed sexual and reproductive decisions and protect their health [9]. Most women with disabilities lack knowledge of sexuality and sexual health, which often limits their capacity to make informed sexual and reproductive health decisions [5, 10]. Lack of access to both formal and informal education on sexuality and sexual health [11] and limited availability of sexual and reproductive health information in disability-appropriate formats such as brailles, audios, and sign interpreters [1] often contribute to poor sexual health knowledge among women with disabilities. Other factors include hesitancy to approach carers on sexual health matters due to societal prejudice and lack of trained or experienced care providers [3, 12]. Meanwhile, limited sexual education and information predispose many women with disabilities to risky sexual behaviours, including having multiple sexual partners and lack of modern contraceptive use [5, 13], thereby increasing their risk of sexual and reproductive health problems.

Despite the importance of contraception in reducing unintended pregnancies, maternal morbidity and mortality, and preventing STIs, including HIV [14], women with disabilities face significant barriers to accessing modern contraceptives, especially in SSA [15, 16]. Access to modern contraceptives among women with disabilities is often limited by a lack of knowledge and education on contraception, poor socio-economic status, lack of disability-friendly healthcare facilities and policies, societal stigma, and discriminatory practices of some healthcare providers [15, 17]. Besides, evidence suggests that women with disabilities have a higher burden of sexual and reproductive health problems due to the lower prevalence of contraceptive use and higher unmet need for modern contraceptives in low- and middle-income countries (LMICs), especially in SSA [18, 19].

The intersection of sexual health knowledge and modern contraceptive use remains an important research area in sexual and reproductive health [20]. Previous studies have shown that good sexual health knowledge increases the use of modern contraceptives due to an improved understanding of the available methods of contraception and how to access them [21, 22]. However, the intersection of sexual health knowledge and modern contraceptive use remains less investigated among women with disabilities in SSA, although they experience greater barriers in accessing sexual health education and information as well as modern contraceptives [15, 18, 23].

Besides, factors such as geographic location play a significant role in access to sexual health information and modern contraceptive use [24, 25]. For instance, previous studies revealed that rural-urban residency could influence sexual health knowledge and contraceptive use of women with disabilities [10, 26]. Nonetheless, the shared impact of spatial patterns of sexual health knowledge and modern contraceptive use among women with disabilities in Africa remain less known. Therefore, this study sought to investigate the spatial patterns of the shared impact of sexual health knowledge and modern contraceptive use among women with disabilities in SSA in order to highlight the geographic areas with increased vulnerability of women with disabilities to sexual and reproductive health problems, at the same time, utilising spatial pattern modelling will inform targeted interventions, that could improve healthcare accessibility, and promotes disability-inclusive policies.

## Data and methods

### Study design and participants

This study utilised the most recent standardised data from the Demographic Health Survey (DHS) conducted in ten African countries. The DHS is a national survey administered in over 90 countries to collect essential health indicators among individuals aged 15–49 [27]. The data for this research were sourced from the women’s and household recode files, which are publicly accessible upon request [28]. The women’s recode file provides information on maternal, sexual, and reproductive health, while disability data were extracted from the household recode file. Only those countries with available disability modules and complete responses for all relevant variables were included in the study [29].

The DHS employs a two-stage sampling process: primary survey units are initially selected, followed by the random selection of participants from clusters within each country. For this study, women aged 15–49 with disabilities were eligible for the disability module, with one woman with a disability from every third selected household included [28, 30]. Participants were identified as having one or more disabilities based on the Washington Group Short Set of Disability Questions, which focuses on difficulties with seeing, hearing, speaking, and walking [31, 32]. The analysis comprised 16,157 weighted women with disabilities who provided complete data on sexual health knowledge, modern contraceptive use, and relevant covariates.

Table 1 details the sample sizes for various countries in Africa. The DHS is recognised as a reliable secondary dataset and has been extensively used in research on sexual and reproductive health, including sexual health knowledge and modern contraceptive use [33, 34]. Data for this study were accessed from the DHS website on February 23, 2024, following a request via https://dhsprogram.com/data/available-datasets.cfm.

**Table 1:** Distribution of weighted eligible DHS in Africa

S/*N*	Country	Survey year	Sample size	Percentage
1	Kenya	2021/2022	1,404	8.69
2	Mauritania	2019/2022	2,436	15.08
3	Rwanda	2019/2020	1,694	10.48
4	Nigeria	2017/2018	629	3.89
5	Mali	2017/2018	947	5.86
6	South Africa	2015/2016	836	5.17
7	Malawi	2015/2016	1,499	9.28
8	Uganda	2015/2016	4,168	25.80
9	Chad	2014/2015	1,553	9.61
10	Congo Dr	2013/2014	991	6.13

### Study variables

#### Study population variable

Women with disabilities were identified using the DHS disability module questionnaire, which was answered by the household head. This questionnaire inquired about the disability status of women aged 15–49, based on the Washington Group’s short set of disability questions. These questions address difficulties in seeing, walking, hearing, remembering, communicating, and self-care, with response options ranging from “No difficulty” to “Cannot do it at all.” This study specifically focused on difficulties in seeing, hearing, speaking, and walking. Women with at least one functional difficulty in these areas from ten African countries were included as women with disabilities [29].

#### Outcome variable

The outcome variable for this study was the use of modern contraceptives among women with disabilities in Africa. Modern contraceptive methods include female and male sterilisation, intrauterine devices, injectables, implants, oral pills, male and female condoms, emergency contraception, and the standard days’ method [35]. Women using any of these methods were classified as modern contraceptive users and coded as “1”. Non-users, coded as “0”, were those who either relied on traditional methods such as the rhythm method, lactational amenorrhea, and withdrawal or had never used contraception. These criteria have been consistently applied in previous research on modern contraceptive use in Africa [34–37].

#### Key independent variable

The key independent variable for this study was sexual health knowledge, measured by questions on “knowledge of ovulation cycle” and “knowledge and use of HIV test kits” in the DHS. Responses were categorised into three levels: Poor, Moderate, and Good. Women with disabilities were classified based on their responses, where “Poor” represented minimal or no sexual health knowledge, “Moderate” indicated a basic understanding, and “Good” denoted comprehensive knowledge [38, 39]. The DHS captures sexual health knowledge to reflect these varying levels of understanding of sexual and reproductive health indicators [32].

### Covariates

The covariates for the analysis were selected based on their relevance to the existing literature [34, 37, 40]. These covariates include age, educational level, marital status, wealth index, mass media exposure, place of residence, community literacy level, community socio-economic level, and community knowledge of modern methods.

For the analysis, the ages of women with disabilities were grouped into three categories: 15–24, 25–34, and 35 and above. Educational levels were classified as no education, primary education, and secondary or higher education. Marital status was divided into never married, currently married, and ever married. Mass media exposure was categorised as either no exposure or exposure to mass media. The place of residence was categorised into rural and urban. Additionally, community literacy level, community socio-economic level, and community knowledge of modern methods were classified as low, medium, and high [34, 37, 40].

### Statistical analyses

#### Shared component model

The shared component model, as adapted from the framework described by Knorr and Best [41], facilitates the estimation of shared patterns that explain the clustering of two or more phenomena—here, sexual health knowledge and modern contraceptive use—as well as the factors unique to each outcome or component. Consider a binary response variable $\:{\gamma\:}_{ijd}$ where $\:{\gamma\:}_{ijd}=0$, indicates that women with disabilities *i* in district *j* has the outcome d, and $\:{\gamma\:}_{ijd}=0$ otherwise, with *i* = 1,2,…,3446, *j* = 1,2,…,30, and *d* = 1, 2. The outcome $\:{\gamma\:}_{ijd}$ ​ is assumed to follow a Bernoulli distribution such that:

1 $$\:{\gamma\:}_{ijd}\sim\:Bernouilli\left({\text{{\rm\:P}}}_{ijd}\right),$$

Where

2 $$\:∱\left({{\upgamma\:}}_{ijd}|{\text{{\rm\:P}}}_{ijd}\right)={\text{{\rm\:P}}}_{ijd}^{{\gamma\:}_{ijd}}(1-{\text{{\rm\:P}}}_{ijd}{)}^{1-{\gamma\:}_{ijd}}$$

3 $$\exp [{\gamma _{ijd}}{\eta _{ijd}} - \log (1 + \exp ({\eta _{ijd}}))]$$

and $\:{\text{{\rm\:P}}}_{ijd}=P\left({\gamma\:}_{ijd}=1\right)\:and\:{\eta\:}_{ijd}$ is the model predictor that describes the modern contraceptive use of women with disabilities who live in district *j*, given that $\:{\eta\:}_{ijd}=logit\left({\text{{\rm\:P}}}_{ijd}\right).\:$Consequently, with the logit link function, the sexual health knowledge of women with disabilities can be linked with selected covariates, which are of different forms, as follows:

4 $$\:logit\left({\text{{\rm\:P}}}_{ijd}\right)=\alpha\:+\omega\:\beta\:+\text{g}\left({V}_{j}\right).\:{\sigma\:}_{d}+{\text{g}}_{d}\left({S}_{j}\right),$$

Where $\:\alpha\:$ represents the intercept shared by the shared outcome variable, $\:\beta\:$ is a vector of coefficients for a shared categorical variable, $\:\omega\:\:$includes parameters specific to individual covariates, $\:\text{g}\left({V}_{j}\right)$captures the shared spatial field affecting the outcomes, $\:{\sigma\:}_{d}\:$controls how the shared variable influences outcome and $\:{\text{g}}_{d}\left({S}_{j}\right)$ denotes the spatial random effects specific to each outcome

The model was implemented using Bayesian inference via the Integrated Nested Laplace Approximation (INLA) method as described by Rue et al. [42]. Priors for $\:{\sigma\:}_{d}$​ were assigned as log-normal distributions with a mean of 0 and precision of 0.1. Weakly informative Gaussian priors were used for the fixed effects and intercepts, with $\:\beta \: \sim N\left( {0,\tau {\:_{\beta \:}}I} \right)$, where $\:{\tau\:}_{\beta\:}$ is the precision.

To model the shared and specific spatial effects, an intrinsic conditional autoregressive (ICAR) prior was employed. The ICAR prior introduces spatial autocorrelation by considering neighbouring districts based on shared boundaries. In its simplest form:

5 $$\:\pi\:\left(s|\tau\:\right)\sim\frac{1}{{Z}_{n}\left(\tau\:\right)}\text{e}\text{x}\text{p}(-\frac{\tau\:}{2}\sum\:{({S}_{i}-{S}_{j})}^{2}$$

where i∼j denotes adjacent districts, *τ* represents the precision parameter controlling spatial smoothness, and $\:{Z}_{n}\left(\tau\:\right)\:$is the normalising constant. Hyperpriors for precision parameters were based on Penalised Complexity (PC) priors, designed to balance the model’s complexity. These priors follow a Type-2 Gumbel distribution with parameters *τ* and *λ* set to 1 and 0.01, respectively [42]. Scaling ensured consistent smoothness across spatial components.

Data preparation and descriptive analysis were conducted using Stata 14, while the Bayesian modelling was implemented with the R-INLA package.

## Results

### Spatial modelling

**Fig. 1:**
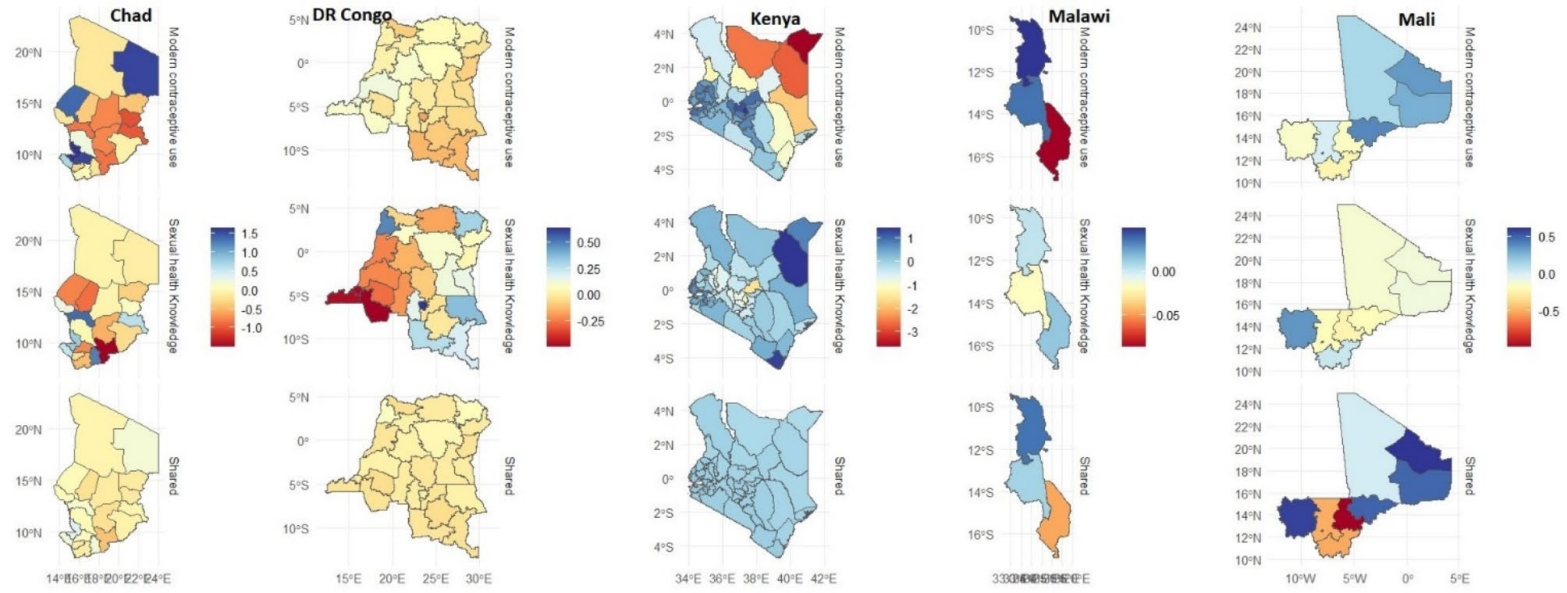
Spatial modelling of sexual health knowledge, modern contraceptive use and shared effects in Chad, DR Congo, Kenya, Malawi and Mali

**Fig. 2:**
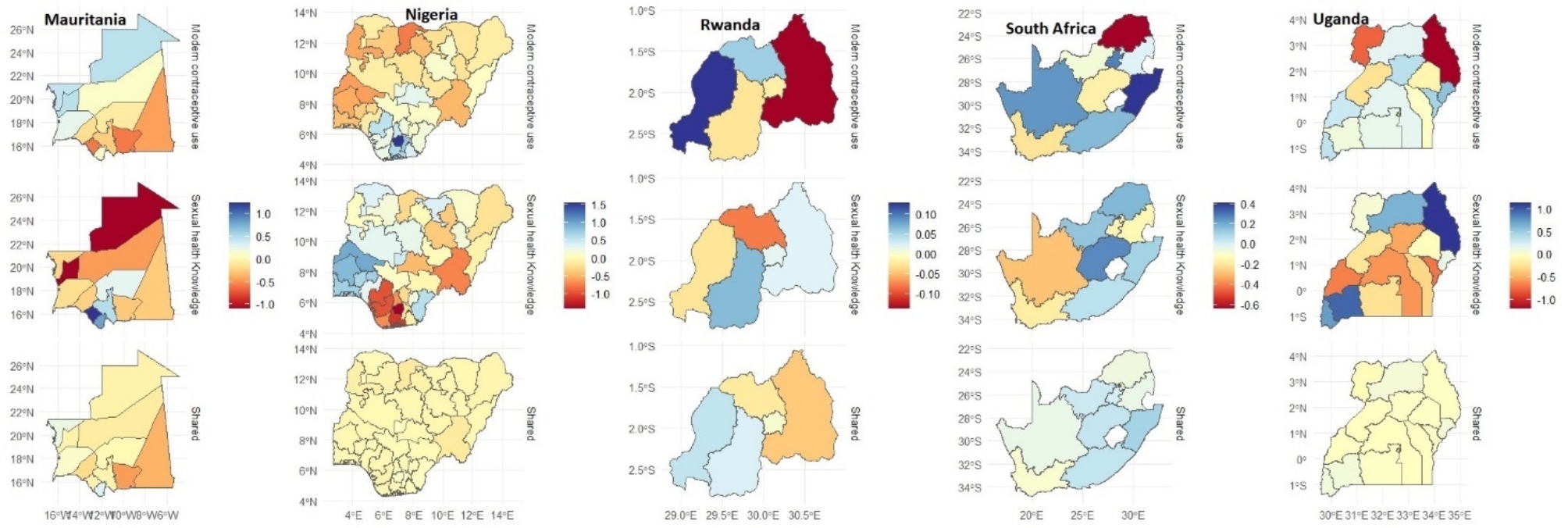
Spatial modelling of sexual health knowledge, modern contraceptive use and shared effects in Mauritania, Nigeria, Rwanda, South Africa and Uganda

Figure 1 presents the spatial effects for Chad, DR Congo, Kenya, Malawi and Mali, respectively, while Fig. 2 presents those for Mauritiana, Nigeria, Rwanda, South Africa and Uganda. The maps show the results for each outcome (specific effects) and the joint (shared) effects that reflect the combined chances for modern contraceptive use and sexual health knowledge.

Results for Chad (Fig. 1) show a higher likelihood of using modern contraceptives among the women with disabilities living in Ennedi Est/Qest, Kanem, and Mayo Quest regions but lower among those in neighbouring Sila and Ouaddai regions. For sexual health knowledge, there appears to be some divergence pattern in the southern part of the country with the likelihood being lower among those living in Moyen Chari but higher for those from Hadjer-Lamis region. The map for the shared effects shows a similar moderate pattern throughout the country. For DR Congo, the maps for modern contraceptive use and the shared effects appear to be similar, reflecting moderate estimates throughout the country. However, the chances for sexual health knowledge are lower among the women with disabilities in the Western part of the country, with those from Kongo Central and Kwango regions having the least likelihood of having sexual health knowledge.

The estimates for Kenya show a lower likelihood for modern contraceptive use in the three neighbouring regions of Mandera, Wajir, and Marsabit but higher among those from the western and southern parts of the country, while for sexual health knowledge, except for Meru, the chances are high in all other parts of the country. The shared effects also show high chances for modern contraceptive and sexual health knowledge throughout Kenya. In Malawi, the results show a higher likelihood of modern contraceptive use among the women with disabilities in the Northern region, followed by those from the Central but lowest among those in the Southern fringe.

The estimates for sexual health knowledge show that the women with disabilities in the Central region have a somewhat lower likelihood of sexual health knowledge, but in the case of the shared effects, there are lower chances for both indicators among those in the Southern region. In the case of Mali, the likelihood of using modern contraceptives is high among those from Gao, Kidal, Mopti, and Tombouctou, but these places have lower chances for sexual health knowledge, which is only high among those living in the Kayes region. However, findings from the shared effects reveal a lower likelihood for both indicators among the women with disabilities residing in Segou but higher for those residing in Kayes, Kidal, Gao, and Mopti.

The results in Fig. 2 show that in the case of Mauritania, the chances of using a modern contraceptive are lower among the women with disabilities in the southern part of the country but higher in Dakhlet Nouadhibou, Tiris Zemour and Inchiri regions, while the chances for sexual health knowledge are only higher in Gorgol, Guidimagha, and Assaba. The map for the shared effects shows slightly lower estimates throughout the country. For Nigeria, except for a few places in the southern part of the country, the chances of using a modern contraceptive by women with disabilities are low in all parts of the country, but for sexual health knowledge, those from the south-western part of the country are more likely to have higher knowledge of sexual health. Like Mauritania, the estimates for the shared effects show a lower likelihood for the combined indicators throughout the country.

In Rwanda, the women with disabilities from the East province have the lowest chances of using modern contraceptives, while those from the West have the highest chances. However, for sexual health knowledge, those from the South, Kigali and East provinces are more likely to know about sexual health, whereas the map for the shared effect shows a slightly higher likelihood among those from the West and South provinces. The maps for South Africa reveal that whereas the women with disabilities from Limpopo were less likely to use modern contraceptives, those from Kwazulu Natal, Eastern Cape, and Northern Cape were more likely.

The findings on sexual health knowledge, on the other hand, show that women with disabilities from the Northern Cape, Western Cape, Mpumalanga, and Gauteng were less likely to have sexual health knowledge. The map for the shared effect is somewhat similar to that of sexual health knowledge, except that the estimated values are slightly lower. Finally, in the case of Uganda, the women with disabilities from Karamoja and West Nile regions have the lowest likelihood of using modern contraceptives, but the women with disabilities from Karamoja, Acholi, Ankole, and Kigezi regions were more likely to have sexual health knowledge. The map for the shared effect is similar to those obtained for Nigeria and Mauritania.

### Weighted country prevalence of modern contraceptive use and sexual health knowledge

**Fig. 3:**
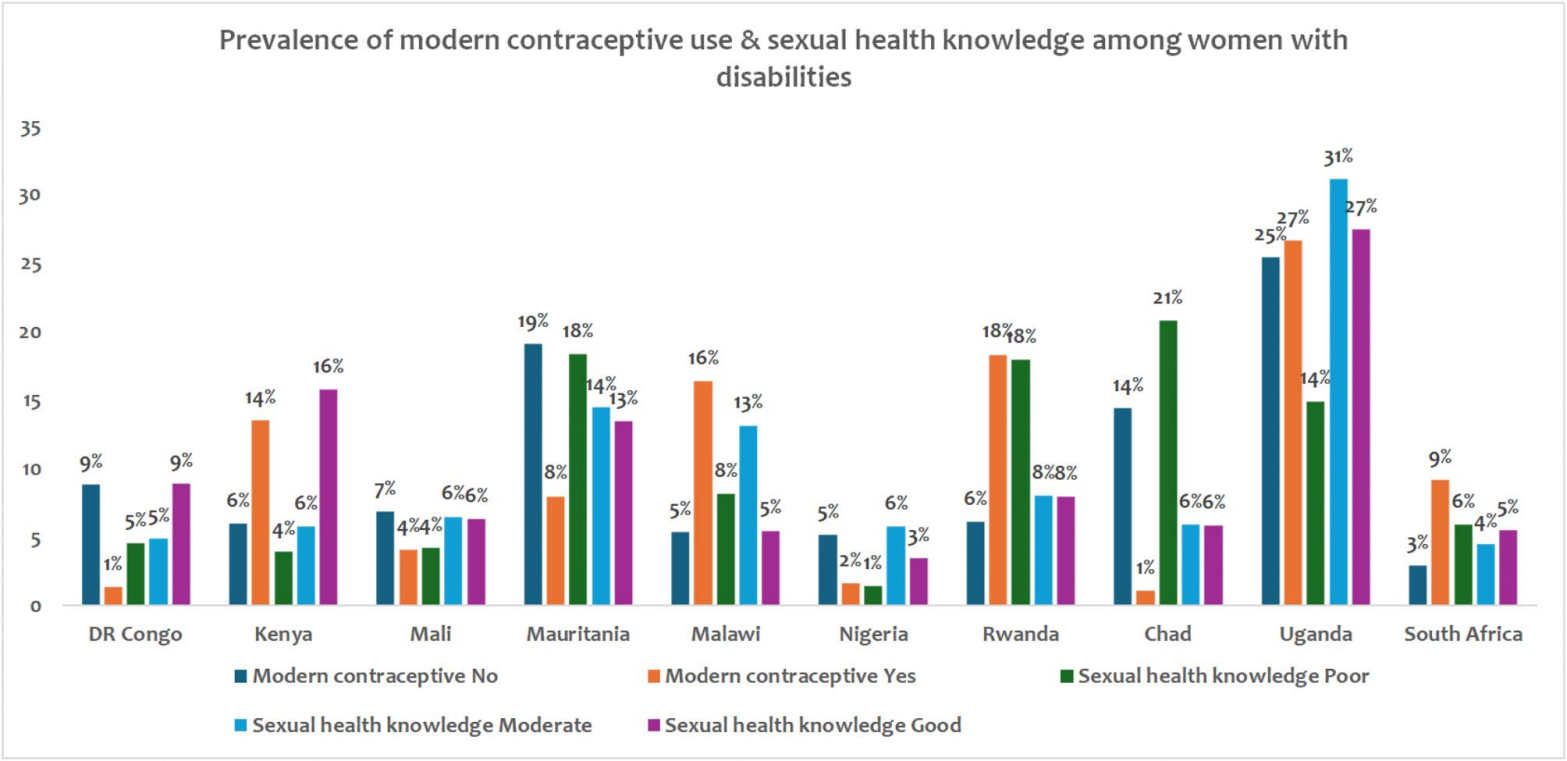
Weighted country-level prevalence of sexual health knowledge and modern contraceptive use among women with disabilities in 10 Africa countries

Figure 3 shows the prevalence of modern contraceptive use and sexual health knowledge among women with disabilities across several countries: DR Congo, Kenya, Mali, Mauritania, Malawi, Nigeria, Rwanda, Chad, Uganda, and South Africa. The data is categorised into modern contraceptive use (Yes and No) and levels of sexual health knowledge (Poor, Moderate, and Good), with the percentages provided for each category.

Overall, modern contraceptive use among women with disabilities was generally low across the countries surveyed. Uganda stands out with the highest prevalence of contraceptive use at 27%, although non-use is also high at 25%, indicating a significant disparity. In contrast, countries like DR Congo (1%) and Chad (1%) have notably low usage rates. Sexual health knowledge varies widely among the countries. Poor sexual health knowledge was prevalent in many countries, especially in Chad (21%) and Mauritania (14%). Conversely, Uganda (31% moderate, 27% good) and Rwanda (8% moderate, 8% good).

Table 2 presents the weighted frequency of modern contraceptive use and sexual health knowledge among women with disabilities, segmented by key explanatory variables. Education significantly influences both modern contraceptive use and sexual health knowledge (X², *p* < 0.001). Women with disabilities with secondary/higher education have higher modern contraceptive use (34.12%) compared to those with no education (11.74%). Additionally, women with disabilities with secondary/higher education have better sexual health knowledge, with 42.65% having good knowledge compared to 16.33% of women with disabilities with no education, whilst marital status also shows a significant relationship with both outcomes (X², *p* < 0.001).

Currently, married women with disabilities have similar rates of contraceptive use (62.13%) compared to ever-married women with disabilities (30.62%). However, never-married women with disabilities have the highest percentage of good sexual health knowledge (7.40%), compared to currently married (61.92%) and ever-married women with disabilities (30.68%). Exposure to mass media is significantly associated with higher contraceptive use and better sexual health knowledge (X², *p* < 0.001). Women with disabilities with access to mass media have higher contraceptive use (78.28%) compared to those without (21.72%). Furthermore, they also have better sexual health knowledge, with 78.26% having good knowledge compared to 21.74% of women with disabilities without access.

For the community level, community literacy level was significantly associated with both contraceptive use and sexual health knowledge (X², *p* < 0.001). Women with disabilities in medium literacy level communities have higher contraceptive use (43.70%) compared to those in low literacy level communities (26.14%). Similarly, good sexual health knowledge is highest among women with disabilities in medium literacy level communities (41.34%) compared to low literacy level communities (23.82%), whilst community socio-economic status showed that women with disabilities from high socio-economic communities have higher contraceptive use (33.53%) and good sexual health knowledge (40.45%) compared to those from low socio-economic communities (57.37% contraceptive use and 54.11% good knowledge).

**Table 2:** Weighted frequency of sexual health knowledge, modern contraceptive use and shared effects among women with disabilities by key explanatory variables

Variables (*n* = 16,517)	Modern contraceptive use (%)		Sexual health knowledge (%)		
	No	Yes	Poor	Moderate	Good
**Education**	$\:{X}^{2}$= *p* < 0.001		$\:{X}^{2}$= *p* < 0.001		
No Education	31.95	11.74	37.99	23.52	16.33
Primary education	42.22	54.14	46.68	51.23	41.03
Secondary/higher	25.61	34.12	15.32	25.25	42.65
Marital status	$\:{X}^{2}$= *p* < 0.001		$\:{X}^{2}$= *p* < 0.001		
Never married	5.37	7.24	6.35	4.73	7.40
Currently married	64.43	62.13	68.10	62.27	61.92
Ever married	30.20	30.62	25.56	33.00	30.68
Mass media	$\:{X}^{2}$= *p* < 0.001		$\:{X}^{2}$= *p* < 0.001		
No	36.59	21.72	42.85	32.06	21.74
Yes	63.41	78.28	57.15	67.94	78.26
Place of residence	$\:{X}^{2}$= 0.086				
Urban	31.62	30.24	25.35	27.54	39.75
Rural	68.37	69.76	74.65	72.46	60.25
**Community literacy level**	$\:{X}^{2}$= *p* < 0.001		$\:{X}^{2}$= *p* < 0.001		
Low	37.08	26.14	44.09	34.19	23.82
Medium	32.47	43.70	31.19	35.73	41.34
High	30.46	30.17	24.72	30.09	34.84
**Community socio-economic level**	$\:{X}^{2}$= *p* < 0.001		$\:{X}^{2}$= *p* < 0.001		
Low	64.27	57.37	67.96	64.38	54.11
Medium	5.14	9.10	6.79	7.33	5.45
High	30.59	33.53	25.24	28.29	40.45

### Estimates modelling for modern contraceptive use and sexual health knowledge by countries

Table 3 shows various factors influencing modern contraceptive use and sexual health knowledge among women with disabilities in Chad, DR Congo, Kenya, Malawi, and Mali.

Education shows a significant influence on both outcomes across several countries. In Chad, primary education (aOR = 13.295, CI: 5.206–33.936) and secondary/higher education (aOR = 3.568, CI: 1.008–12.634) significantly increase the odds of modern contraceptive use, with secondary/higher education also enhancing sexual health knowledge (aOR = 13.653, CI: 7.100-26.340). In Kenya, both primary education (aOR = 1.788, CI: 1.243–2.572) and secondary/higher education (aOR = 1.556, CI: 1.028–2.355) increase the odds of contraceptive use, and similarly, primary (aOR = 1.826, CI: 1.152–2.903) and secondary/higher education (aOR = 5.061, CI: 2.653–9.677) significantly boost sexual health knowledge. In Mali, education positively influences contraceptive use, with primary education (aOR = 2.236, CI: 1.364–3.664) and secondary/higher education (aOR = 2.303, CI: 1.450–3.658) showing increased odds.

Marital status presents varying impacts across countries. In Chad, currently married (aOR = 3.733, CI: 1.871–7.452) and previously married women with disabilities (aOR = 8.747, CI: 4.247–18.020) have higher odds of better sexual health knowledge but lower odds of using modern contraceptives (currently married aOR = 0.275, CI: 0.040–1.891; previously married aOR = 0.028, CI: 0.041–1.929). In DR Congo, previously married women with disabilities show higher odds for sexual health knowledge (aOR = 2.650, CI: 1.115–6.301). In Kenya, marital status positively impacts sexual health knowledge, with currently married (aOR = 2.855, CI: 1.652–4.930) and previously married women with disabilities (aOR = 7.168, CI: 3.493–14.717) showing higher odds. In Malawi, while previously married women with disabilities have lower odds of using contraceptives (aOR = 0.546, CI: 0.310–0.962), currently married women with disabilities show better sexual health knowledge (aOR = 2.527, CI: 1.410–4.526). In Mali, currently married (aOR = 0.229, CI: 0.118–0.443) and previously married women with disabilities (aOR = 0.140, CI: 0.041–0.476) have lower odds of using modern contraceptives, but previously married women with disabilities show higher odds of better sexual health knowledge (aOR = 5.251, CI: 1.501–18.481).

Place of residence significantly influences outcomes. In Chad, rural residence increases the odds of using modern contraceptives (aOR = 6.305, CI: 1.870-22.351). Conversely, in Kenya and Mali, rural residence decreases the odds (Kenya: aOR = 0.524, CI: 0.384–0.715; Mali: aOR = 0.282, CI: 0.147–0.535). However, in Mali, rural residence increases the odds of better sexual health knowledge (aOR = 2.196, CI: 1.152–4.157).

Community literacy levels show varied effects. In Chad, higher community literacy levels significantly increase the odds of using modern contraceptives (medium: aOR = 6.305, CI: 1.870-22.351; high: aOR = 3.882, CI: 1.107–13.673). In Kenya, medium literacy levels decrease the odds of contraceptive use (aOR = 0.684, CI: 0.476–0.982). In DR Congo, community literacy levels positively affect sexual health knowledge, with primary education increasing the odds (aOR = 2.467, CI: 1.545–3.943).

Community socioeconomic status influences outcomes differently. In Mali, high socioeconomic status decreases the odds of using modern contraceptives (aOR = 0.523, CI: 0.284–0.960). In Malawi, high socioeconomic status increases the odds of better sexual health knowledge (aOR = 2.093, CI: 1.225–3.574).

Age impacts outcomes, with older women with disabilities generally showing higher odds for both modern contraceptive use and sexual health knowledge. In Chad, women with disabilities aged 25–34 have higher odds of using modern contraceptives (aOR = 4.553, CI: 1.160-17.859). In Kenya, women with disabilities aged 35 and above have lower odds of using modern contraceptives (aOR = 0.646, CI: 0.461–0.904). In Mali, older women with disabilities have higher odds of using modern contraceptives (aOR = 2.503, CI: 1.531–4.094).

**Table 3:** Estimated odds ratio for and 95% credible intervals specific for contraceptive use and sexual health knowledge for the categorical variables for Chad, congo, Kenya, Malawi and Mali among women with disabilities

Modern contraceptive use					
Variable	Chad	DR Congo	Kenya	Malawi	Mali
**Education**					
No Education Primary Secondary/Higher	1 13.295 (5.206, 33.936) 3.568 (1.008, 12.634)	1 0.647 (0.255, 1.641) 2.182 (0.886, 5.376)	1 1.788 (1.243, 2.572) 1.556 (1.028, 2.355)	1 1.392 (1.018, 1.903) 1.343 (0.863, 2.090)	1 2.236 (1.364, 3.664) 2.303 (1.450, 3.658)
**Marital Status**					
Never married Currently married Previously married	1 0.275 (0.040, 1.891) 0.028 (0.041, 1.929)	1 0.779 (0.284, 2.144) 1.048 (0.367, 2.996)	1 1.271 (0.851, 1.892) 0.978 (0.621, 1.541)	1 0.934 (0.553, 1.578) 0.546 (0.310, 0.962)	1 0.229 (0.118, 0.443) 0.140 (0.041, 0.476)
**Place of residence**					
Urban Rural	1 6.305 (1.870, 22.351)	1 0.421 (0.196, 0.903)	1 0.524 (0.384, 0.715)	1 1.052 (0.670, 1.582)	1 0.282 (0.147, 0.535)
**Community literacy level**					
Low Medium High	1 6.305 (1.870, 22.351) 3.882 (1.107, 13.673)	1 1.223 (0.507, 2.955) 0.903 (0.324, 2.513)	1 0.684 (0.476, 0.982) -	1 0.943 (0.717, 1.240) 1.212 (0.897, 1.639)	1 0.911 (0.551, 0.505) 0.963 (0.590, 1.571)
**Community Socioeconomic Status**					
Low Medium High	1 1.120 (0.120, 11.019) 5.545 (1.606, 20.254)	1 - 1.109 (0.529, 2.322)	1 - 0.673 (0.469, 0.966)	1 1.270 (0.926, 1.740) 1.023 (0.726, 1.442)	1 0.938 (0.486, 1.809) 0.523 (0.284, 0.960)
**Age**					
15–24 25–34 35 and above	1 4.553 (1.160, 17.859) 3.916 (0.993,15.439)	1 1.134 (0.551, 2.332) 1.289 (0.610, 2.723)	1 1.223 (0.921, 1.624) 0.646 (0.461, 0.904)	1 1.125 (0.861,1.471) 1.016 (0.731, 1.413)	1 1.476 (0.941, 2.315) 2.503 (1.531, 4.094)
**Sexual health knowledge**					
**Education**					
No Education Primary Secondary/Higher	1 1.272 (0.874, 1.849) 13.653 (7.100, 26.340)	1 2.467 (1.545, 3.943) 4.363 (2.424, 7.877)	1 1.826 (1.152, 2.903) 5.061 (2.653, 9.677)	1 1.367 (0.965, 1.937) 2.093 (1.225, 3.574)	1 0.739 (0.447, 1.222) 1.353 (0.799, 2.293)
**Marital Status**					
Never married Currently married Previously married	1 3.733 (1.871, 7.4520) 8.747 (4.247, 18.020)	1 1.240 (0.545, 2.819) 2.650 (1.115, 6.301)	1 2.855 (1.652, 4.930) 7.168 (3.493, 14.717)	1 2.527 (1.410, 4.526) 1.679 (0.900, 3.137)	1 1.260 (0.675, 2.363) 5.251 (1.501, 18.481)
**Place of residence**					
Urban Rural	1 0.462 (0.302, 0.708)	1 0.437 (0.235, 0.814)	1 0.674 (0.433, 1.048)	1 0.989 (0.617, 1.587)	1 2.196 (1.152, 4.157)
**Community literacy level**					
Low Medium High	1 3.733 (1.871, 7.452) 8.747 (4.247, 18.020)	1 0.471 (0.291, 0.760) 0.670 (0.344,1.301)	1 2.138 (1.369, 3.340) -	1 1.118 (0.818, 1.528) 1.628 (1.133, 2.340)	1 1.320 (0.801, 2.175) 0.625 (0.387, 1.009)
**Community Socioeconomic Status**					
Low Medium High	1 1.138 (0.683, 1.900) 0.897 (0.561, 1.434)	1 - 0.675 (0.338, 1.349)	1 - 1.684 (0.845, 3.352)	1 1.393 (0.948, 2.046) 0.846 (0.572, 1.252)	1 1.396 (0.730, 2.669) 2.050 (1.063, 3.951)
**Age**					
15–24 25–34 35 and above	1 1.717 (1.192, 2.474) 1.617 (1.141, 2.292)	1 0.694 (0.426, 1.131) 1.315 (0.750, 2.305)	1 1.291 (0.855, 1.950) 1.314 (0.819, 2.108)	1 0.945 (0.688, 1.297) 1.030 (0.699, 1.517)	1 1.798 (1.196, 2.703) 1.606 (1.019, 2.533)

Table 4 shows how modern contraceptive use and sexual health knowledge were significant with other variables among women with disabilities in Mauritania, Nigeria, Rwanda, South Africa, and Uganda. The following analysis integrates these findings and highlights common themes across countries.

Education consistently emerges as a critical factor. In Mauritania, secondary/higher education significantly increases the odds of modern contraceptive use (aOR = 1.724, CI: 1.220–2.438), while in Nigeria and Uganda, it significantly boosts sexual health knowledge (Nigeria: aOR = 3.367, CI: 2.466–4.595; Uganda: aOR = 2.979, CI: 2.116–4.195). Similarly, in Rwanda and South Africa, secondary/higher education enhances sexual health knowledge (Rwanda: aOR = 1.930, CI: 1.319–2.822; South Africa: aOR = 2.253, CI: 1.391–3.650).

Marital status shows an impact across several countries. In Mauritania and Nigeria, currently married and previously married women with disabilities have significantly lower odds of using modern contraceptives (Mauritania: currently married aOR = 0.271, CI: 0.160–0.459; previously married aOR = 0.001, CI: 0.000-0.011; Nigeria: currently married aOR = 0.266, CI: 0.132–0.537; previously married aOR = 0.237, CI: 0.098–0.571). However, in Rwanda and South Africa, currently married and previously married women with disabilities have higher odds of using modern contraceptives (Rwanda: currently married aOR = 1.921, CI: 1.317–2.801; previously married aOR = 1.763, CI: 1.230–2.527; South Africa: currently married aOR = 1.781, CI: 1.195–2.656; previously married aOR = 2.345, CI: 1.571-3.500). Marital status also significantly boosts sexual health knowledge in Nigeria and Uganda, where currently and previously married women with disabilities show higher odds (Nigeria: previously married aOR = 9.310, CI: 2.735–31.868; Uganda: currently married aOR = 1.848, CI: 1.307–2.613; previously married aOR = 2.027, CI: 1.453–2.829).

Place of residence shows mixed effects. In Mauritania and Uganda, rural residence significantly decreases the odds of using modern contraceptives (Mauritania: aOR = 0.421, CI: 0.288–0.616; Uganda: aOR = 0.606, CI: 0.496–0.740). Conversely, in South Africa, rural residence increases the odds (aOR = 1.568, CI: 1.092–2.258). Rural residence also impacts sexual health knowledge negatively in Rwanda (aOR = 0.504, CI: 0.372–0.684) and positively in South Africa (aOR = 1.568, CI: 1.092–2.258).

Community literacy level affects outcomes in several contexts. In Nigeria, medium community literacy decreases the odds of modern contraceptive use (aOR = 0.370, CI: 0.184–0.742). High community literacy significantly increases sexual health knowledge in Nigeria (aOR = 2.522, CI: 1.023–6.198) and Rwanda (aOR = 1.361, CI: 1.030–1.797).

Community socioeconomic status is significant in South Africa, where high socioeconomic status increases sexual health knowledge (aOR = 2.917, CI: 1.587–5.366). In Nigeria, medium socioeconomic status boosts modern contraceptive use (aOR = 3.574, CI: 1.387–9.220).

Age impacts both modern contraceptive use and sexual health knowledge. In Mauritania, older age groups have lower odds of using modern contraceptives (25–34 years: aOR = 0.690, CI: 0.492–0.969; 35 and above: aOR = 0.650, CI: 0.460–0.917). In Uganda, older age groups show higher odds (25–34 years: aOR = 1.221, CI: 1.031–1.446; 35 and above: aOR = 1.233, CI: 1.023–1.485). For sexual health knowledge, older age groups have higher odds in Uganda (25–34 years: aOR = 1.678, CI: 1.344–2.094; 35 and above: aOR = 1.318, CI: 1.041–1.671), while in South Africa, 35 and above increases odds (aOR = 1.991, CI: 1.210–3.277).

**Table 4:** Estimated odds ratio for and 95% credible intervals specific for contraceptive use and sexual health knowledge for the categorical variables for Mauritania, Nigeria, Rwanda, South Africa, and Uganda among women with disabilities

Modern contraceptive use					
Variable	Mauritania	Nigeria	Rwanda	South Africa	Uganda
Education					
No Education Primary Secondary/Higher	1 1.338 (0.989, 1.811) 1.724 (1.220, 2.438)	1 0.603 (0.270, 1.349) 1.013 (0.496, 2.071)	1 1.128 (0.843, 1.508) 1.250 (0.857, 1.823)	1 0.813 (0.436, 1.513) 0.881 (0.555, 1.397)	1 1.158 (0.958, 1.401) 1.158 (0.907, 1.479)
**Marital Status**					
Never married Currently married Previously married	1 0.271 (0.160, 0.459) 0.001 (0.000, 0.011)	1 0.266 (0.132, 0.537) 0.237 (0.098, 0.571)	1 1.921 (1.317, 2.801) 1.763 (1.230, 2.527)	1 1.781 (1.195, 2.656) 2.345 (1.571, 3.500)	1 0.502 (0.380, 0.662) 0.367 (0.281, 0.480)
**Place of residence**					
Urban Rural	1 0.421 (0.288, 0.616)	1 0.623 (0.337, 1.152)	1 1.001 (0.747, 1.354)	1 1.568 (1.092, 2.258)	1 0.606 (0.496, 0.740)
**Community literacy level**					
Low Medium High	1 0.993 (0.704, 1.399) 1.167 (0.824, 1.655)	1 0.370 (0.184, 0.742) 0.520 (0.261, 1.033)	1 0.768 (0.596, 0.990) 0.901 (0.679, 1.197)	1 1.186 (0.863, 1.630) -	1 1.098 (0.930, 1.296) 1.199 (0.978, 1.471)
**Community Socioeconomic Status**					
Low Medium High	1 - 0.890 (0.625, 1.265)	1 3.574 (1.387, 9.220) 1.255 (0.601, 2.619)	1 1.074 (0.814, 1.419) 0.856 (0.622, 1.177)	1 - 0.849 (0.514, 1.404)	1 0.963 (0.734, 1.264) 1.183 (0.981, 1.425)
**Age**					
15–24 25–34 35 and above	1 0.690 (0.492, 0.969) 0.650 (0.460, 0.917)	1 0.898 (0.554, 1.457) 1 (0.002, 493.176)	1 1.116 (0.803, 1.551) 0.586 (0.417, 0.822)	1 1.033 (0.721, 1.481) 0.553 (0.361, 0.845)	1 1.221 (1031, 1.446) 1.233 (1.023, 1.485)
**Sexual health knowledge**					
**Education**					
No Education Primary Secondary/Higher	1 1.356 (1.089, 1.688) 3.367 (2.466, 4.595)	1 1.561 (0.632, 3.903) 2.057 (0.887, 4.829)	1 1.195 (0.898, 1.591) 1.930 (1.319, 2.822)	1 1.589 (0.834, 3.026) 2.253 (1.391, 3.650)	1 1.667 (1.329, 2.091) 2.979 (2.116, 4.195)
**Marital Status**					
Never married Currently married Previously married	1 0.920 (0.610, 1.387) 1.687 (1.020, 2.790)	1 3.545 (1.635, 7.691) 9.310 (2.735, 31.868)	1 1.232 (0.842, 1.803) 1.336 (0.929, 1.922)	1 1.217 (0.784, 1.890) 0.889 (0.591, 1.337)	1 1.848 (1.307, 2.613) 2.027 (1.453, 2.829)
**Place of residence**					
Urban Rural	1 0.714 (0.532, 0.958)	1 2.100 (0.974, 4.548)	1 0.504 (0.372, 0.684)	1 1.049 (0.716, 1.530)	1 1.060 (0.806, 1.394)
**Community literacy level**					
Low Medium High	1 0.965 (0.750, 1.240) 1.307 (0.989, 1.726)	1 0.791 (0.370, 1.684) 2.522 (1.023, 6.198)	1 1.211 (0.946, 1.550) 1.361 (1.030, 1.797)	1 1.014 (0.723, 1.422) -	1 1.027 (0.823, 1.448) 1.092 (0.823, 1.448)
**Community Socioeconomic Status**					
Low Medium High	1 0.789 (0.582, 1.068) -	1 0.346 (0.090, 1.321) 0.609 (0.248, 1.494)	1 0.967 (0.739, 1.266) 0.884 (0.644, 1.213)	1 - 2.917 (1.587, 5.366)	1 2.358 (1.481, 3.756) 1.244 (0.960, 1.613)
**Age**					
15–24 25–34 35 and above	1 1.564 (1.174, 2.084) 1.465 (1.098, 1.954)	1 2.073 (1.043, 4.120) 1 (0.002, 493.176)	1 0.870 (0.626, 1.208) 0.961 (0.683, 1.352)	1 0.649 (0.442, 0.951) 1.991 (1.210, 3.277)	1 1.678 (1.344, 2.094) 1.318 (1.041, 1.671)

## Discussion

This study revealed low sexual health knowledge (ranging from 3% in Nigeria to 27% in Uganda) and modern contraceptive use (ranging from 1% in DR Congo and Chad to 27% in Uganda) among women with disabilities across the African countries surveyed. The spatial patterns showed diverse intra-country and inter-country disparities of sexual health knowledge and modern contraceptive use among the women, with a generally lower shared impact observed in Mauritania, Nigeria, Uganda, Chad, and DR Congo relative to Kenya, Malawi, Mali, South Africa, and Rwanda. Factors that influence sexual health knowledge and modern contraceptive use among women with disabilities include education, marital status, place of residence, community literacy level, community socio-economic status, and age.

Findings from the current study support previous studies which reported low sexual health knowledge [5, 39] and low prevalence of modern contraceptive use [17, 43, 44] among women with disabilities in Africa. For instance, Ayiga and Kigozi [44] found that only 26.1% of women with disabilities of reproductive age in Uganda have ever used contraception, whilst Tenaw et al. [17] reported a pooled prevalence of 21.6% of contraceptive use among women with disabilities in SSA. Meanwhile, studies in the United States [70.1%] [45] and India [73.0%] [46] showed a high prevalence of contraceptive use among women with disabilities, which was also comparable to the non-disabled population in those countries.

The poor sexual health knowledge and low prevalence of contraceptives recorded in the present study suggest that women with disabilities in Africa remain highly predisposed to sexual and reproductive health problems such as unintended pregnancies and STIs, including HIV. Therefore, there is a need for enhanced public health measures to improve sexual health knowledge and promote the use of modern contraceptives among women with disabilities in Africa.

Meanwhile, we found that age influenced sexual health knowledge and modern contraceptive use among women with disabilities, with older women showing higher odds of both sexual health knowledge and modern contraceptive use in most countries. Previous studies revealed that women with disabilities of higher age are more likely to use modern contraceptives than younger ones [15, 23]. However, in Ethiopia, Tessema et al. [43] reported lower odds of contraceptive use among women with disabilities with higher age (35 years and above) than the younger ones (below 30 years).

Available evidence shows that sexual health knowledge among women with disabilities is often inaccurate, limited in some areas, or contains misconceptions, which affect their sexual and reproductive health behaviour [39], including the use of contraceptives. In support of findings from previous studies [43, 47–49], this study found increased odds of good sexual health knowledge and modern contraceptive use among women with disabilities with formal education relative to those with no education.

Understandably, education improves individuals’ knowledge and access to information on sexual health and contraception, demystifying the negative perceptions and beliefs associated with sexuality and contraceptive use among women with disabilities [17, 44]. Interestingly, we observed that both women with primary education and those with secondary/higher education in South Africa had lower odds of modern contraceptive use relative to those with no education, highlighting the need for further studies in South Africa to ascertain the current observation. Another plausible reason for this could be the socio-cultural belief on contraceptive use in the country [50].

Meanwhile, we found that an increase in community literacy level was generally associated with increased odds of sexual health knowledge among women with disabilities, although its impact on the use of modern contraceptives was diverse. Plausibly, whereas women in communities with high literacy levels are more likely to have increased access to information and resources which could improve their sexual health awareness [51], other structural and systemic barriers may curtail their ability to access and use modern contraceptives.

Further, although there is a consensus on the association between marital status and sexual health knowledge [52, 53], the influence of marital status on modern contraceptive use remains largely inconclusive [54, 55]. Meanwhile, the current findings revealed the diverse impact of marital status on sexual health knowledge and modern contraceptive use among women with disabilities.

For instance, although both currently and previously married women in Chad had higher odds of good sexual health knowledge, they reported lower odds using modern contraceptives. However, in South Africa and Rwanda, currently, married women have higher odds of good sexual health knowledge as well as modern contraceptive use. Structural, socio-economic and cultural differences across the countries could explain the current findings. For instance, in countries like Chad, where there is limited promotion of sexual and reproductive rights of women [56], having good sexual health knowledge may not be enough to promote the use of modern contraceptives among women with disabilities.

We also found varied associations between place of residence and sexual health knowledge, as well as modern contraceptive use. For instance, although women with disabilities in rural Mauritania had lower odds of both sexual health knowledge and modern contraceptive use, those in rural Nigeria had lower odds of modern contraceptive use despite having good sexual health knowledge. The current findings largely agree with findings from previous studies that suggested low use of modern contraceptives among women with disabilities in rural settings compared to those in urban settings [44, 57]. Structural barriers to accessing contraceptives, limited access to healthcare facilities, as well as poor socio-economic status are often implicated in the low utilisation of modern contraceptives among women with disabilities in rural areas in Africa [44, 57, 58].

Although previous studies associated poor sexual knowledge [59, 60] and limited use of modern contraceptives [61] with women with disabilities from low socio-economic communities, we observed an inconsistent association with regard to the influence of community socioeconomic status on sexual health knowledge and modern contraceptive use. For instance, in Mali, high socio-economic status was associated with increased sexual health knowledge but decreased odds of using modern contraceptives among women with disabilities. However, high socio-economic status was associated with increased odds of modern contraceptive use but decreased odds of sexual health knowledge among women with disabilities in Chad. These disparities may be influenced by cultural norms, healthcare accessibility, and regional differences in reproductive health policies [61].

### Strengths and limitations

This study used the most recent DHS datasets of ten African countries to investigate the shared impact of sexual health knowledge and modern contraceptive use among women with disabilities in Africa. However, the study has some limitations. First, both sexual health knowledge and the use of modern contraceptives among women with disabilities could be influenced by the type of disability, such as difficulties in seeing, hearing, speaking or walking [17, 45, 51]; the current analysis did not segregate respondents by disability type. Second, whilst DHS data are generally collected through self-reporting, the disability-related information is obtained from household heads, which could introduce bias in reporting household members’ use of modern contraceptives and sexual health knowledge. Also, due to the cross-sectional nature of this study, we could only report on the associations between the variables of interest and not make causal inferences. Finally, the data source used did not include variables on social or medical models, which could be of important use in providing more information about the barriers faced by women with disabilities in Africa.

### Policy and practical implications

The current findings have significant practical and policy implications. First, the findings show that sexual health knowledge and modern contraceptive use among women with disabilities was generally low across the African countries surveyed. Thus, there is a need for policymakers to heighten efforts aimed at improving sexual health knowledge and promoting the use of modern contraceptives among women with disabilities in Africa.

Second, although the implementation of general measures to improve sexual health knowledge and modern contraceptive use among women with disabilities remains relevant, the spatial analysis revealed areas with a higher burden of the phenomenon, which could guide policymakers towards designing and implementing targeted interventional programmes. Besides, the spatial patterns could also guide future researchers in conducting further studies to examine the peculiarities of the areas with low sexual health knowledge and modern contraceptive use among women with disabilities in order to inform policy decisions.

Additionally, contrary to findings from previous studies [5, 13], the current study revealed that having good sexual health knowledge does not necessarily promote the use of modern contraceptives among women with disabilities. Thus, efforts to promote the use of modern contraceptives among women with disabilities should pay much attention to the structural, economic, and socio-cultural factors that hinder the access and utilisation of modern contraceptives among women with disabilities in Africa.

### Conclusion and recommendations

Sexual health knowledge and modern contraceptive use among women with disabilities in Africa remain low, albeit with varied intra-country and inter-country spatial disparities. Therefore, spatial areas with low sexual health knowledge and modern contraceptive use should be given more attention when implementing measures to promote the use of modern contraceptives among women with disabilities, with much focus on women with disabilities with no education, the unmarried, those who reside in rural areas, those from communities with low literacy level and low socio-economic status, and those with younger age.

Although promoting sexual health knowledge among women with disabilities could influence their use of modern contraceptives to some extent, policymakers need to pay much attention to the structural, economic, and socio-cultural barriers that hinder access and utilisation of modern contraceptives among women with disabilities. In countries with the lowest prevalence, community-based outreach programs, subsidised contraceptive distribution, and inclusive reproductive health education should be prioritised. Additionally, integrating disability-friendly sexual and reproductive health services within primary healthcare systems, strengthening policy frameworks to ensure accessibility, and engaging community and religious leaders to reduce stigma could enhance uptake. Such measures could significantly contribute towards the realisation of the 2030 Sustainable Development Goal agenda of “leaving no one behind”.

## Data Availability

The datasets utilised in this study can be accessed at https://dhsprogram.com/data/available-datasets.cfm.

## Abbreviations

DR Congo: Democratic Republic of Congo
STIs: Sexually Transmitted Infections
HIV: Human Immunodeficiency Virus
SSA: Sub-Saharan Africa
DHS: Demographic and Health Surveys
LMICs: Low- and Middle-Income Countries
ICAR: Intrinsic Conditional Autoregressive
PC: Penalised Complexity
INLA: Integrated Nested Laplace Approximation
